# Aligned Fibronectin Microenvironment Temporally Facilitates Profibrotic Fibroblast Activation via Integrin α5β1

**DOI:** 10.1002/advs.202600047

**Published:** 2026-03-24

**Authors:** Doğuhan Beyatlı, Mika Brown, George‐Radu Romanescu, Seungkuk Ahn

**Affiliations:** ^1^ UCD Charles Institute of Dermatology School of Medicine University College Dublin Dublin Ireland; ^2^ Conway Institute of Biomolecular and Biomedical Research University College Dublin Dublin Ireland

**Keywords:** aligned nanofiber, fibroblast, fibronectin, fibrosis, integrin, mechanobiology

## Abstract

Excessive, aligned nanofibrous extracellular matrix deposition is a main feature of fibrosis, a pathology indicative of poor prognosis in various chronic diseases. Fibronectin is one of the major nanofibrous extracellular matrix proteins that contribute to tissue homeostasis and repair via its cell surface receptors, integrins. Although mechanosensing of aligned fibronectin by fibroblasts is also implicated in the initiation and progression of fibrogenesis, the mechanisms involved remain uncertain. To investigate how profibrotic cell responses and fibrotic tissue development are triggered through these interactions in vitro, we utilized fibroblasts expressing fibronectin‐binding integrins (αV‐class and α5β1 integrins) cultured on fibronectin‐coated electrospun nanofibers with random or aligned networks. During early adhesion, fibroblasts employ a specific integrin, α5β1 integrin, to respond to the aligned nanofibrous fibronectin microenvironment by strengthening cell and F‐actin alignments, nascent focal adhesion, and alpha‐smooth muscle actin expression that are signatures of early pro‐fibrotic activities. Interestingly, α5β1 integrin‐mediated fibronectin alignment sensing continues to form fibrotic tissues at a later stage associated with higher alignment, greater alpha‐smooth muscle actin expression, and extensive extracellular matrix deposition. This mechanistic insight paves the way to better understand the role of fibronectin and its properties in pathophysiology, representing a new target for potential applications in drug discovery.

## Graphical abstract

Compared to a healthy random nanofibrous fibronectin microenvironment, a fibrotic aligned nanofibrous fibronectin microenvironment employs a specific integrin, α5β1 integrin, to activate early pro‐fibrotic fibroblast behaviors and later fibrotic fibroblast tissue formation with cell/F‐actin alignment, α‐SMA expression, and cell‐driven aligned extracellular matrix deposition. This finding unravels a new mechanistic link between fibronectin and fibrosis.

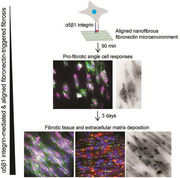

## 1 Introduction

Fibrosis impacts billions of patients with an immense associated financial burden. In 2019, fibrosis was involved in 35.4% of all deaths globally [1]. Fibrosis is characterized by an excessive production of extracellular matrix (ECM), such as collagens and affects all organs [2, 3]. Another key feature of fibrosis is a highly aligned nanofibrous ECM microenvironment leading to dense and stiff fibrotic tissue in contrast to randomly oriented and soft healthy tissue [4]. Despite the severity and urgency of this pathology, the early diagnosis of fibrosis and the development of effective anti‐fibrotic drugs have been challenging due to a lack of mechanistic insights and thus proper targets [5]. Therefore, there is a great need for better understanding mechanisms behind tissue fibrosis at various molecular and structural levels.

Fibronectin (FN) is one of the major ECM proteins that plays a crucial role in developing, maintaining, and remodeling fibrous ECM microenvironments [6]. In fibrotic tissues, FN is highly expressed and required for activating collagen matrix deposition and corresponding pro‐fibrotic cell fates [7, 8, 9]. Cells sense and respond to FN via cell membrane receptors called integrins [10]. Integrins are heterodimeric proteins consisting of 18 α and 8 β subunits making 24 different integrins in mammals. Among various integrins in mammalian cells, αV class and α5β1 integrins are primary sensors of FN [11]. Currently, the FN‐binding αV‐class and α5β1 integrins are understood to crosstalk with each other [12] and drive fibrosis via integrin‐mediated signaling pathways including fibroblast to myofibroblast transition [13] as well as enhanced focal adhesion and stress fiber formation (e.g., α‐smooth muscle actin (α‐SMA)) for generating contractile forces in fibrotic tissues [14, 15]. Although there is a rough consensus regarding the role of FN and FN‐binding integrins in fibrosis, it remains elusive which type of, when, and how integrins mechanosense FN cues at a molecular level and early stage of fibrosis development. Previous in vitro fibrosis studies have predominantly focused on developing in vitro collagen models [16, 17] and understanding the role of collagens in fibrosis [18, 19, 20, 21]. Unlike collagens, fibronectin has been understudied due to technical difficulties in developing in vitro FN‐based models and investigating FN‐binding integrins related to fibrosis.

In this study, we employ random and aligned nanofibers mimicking healthy and fibrotic tissue microstructure, respectively. These nanofibers are coated with FN and tested with engineered fibroblasts only expressing FN‐binding (αV‐class and α5β1) integrins to characterize how nanofibrous FN alignment interacts with specific integrins to initiate and progress fibrosis. We find that fibroblasts use a specific FN‐binding integrin (α5β1 integrin) to sense the aligned nanofibrous FN microenvironment to activate the initial profibrotic fibroblast response and late fibrotic tissue formation with extensive ECM deposition. Our results suggest a crucial role of α5β1 integrins and aligned FN microenvironments in fibrogenesis that can provide a novel mechanobiological insight and a potential target for fibrosis.

## 2 Results

### 2.1 α5β1 Integrins Drive Early Single Cell Morphology, Actin Orientation, and Focal Adhesion in Response to the Aligned Nanofibrous FN Microenvironment

**FIGURE 1:**
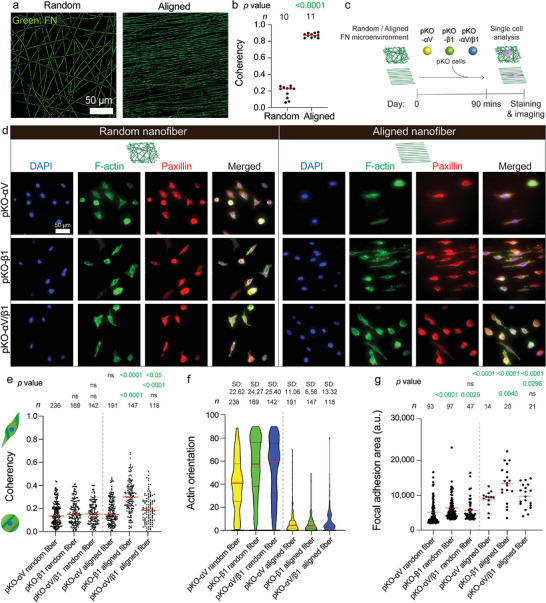
Cell alignment, F‐actin orientation, and focal adhesion formation of FN‐binding integrin‐expressing pKO fibroblasts cultured on the random and aligned nanofibrous FN microenvironments. (a, b) Characterization of random and aligned nanofibrous FN microenvironments with (a) FN (green) distribution and (b) fiber alignment analysis from three biological replicates. Dots represent coherency (or alignment) of individual nanofiber scaffolds and red bars the median. (c–g) Early cell alignment, F‐actin orientation, and focal adhesion of pKO fibroblasts. c) Schematic illustration of experiments. (d–g) Representative immunofluorescence images of pKO fibroblasts grown on the random and aligned FN microenvironments after 90 min of culture with analysis of (e) single cell coherency, (f) F‐actin orientation distribution, (g) focal adhesion area. Blue, green, and red in (d) indicate DAPI, F‐actin, and paxillin, respectively. In (e) and (g), top row *p*‐values compare random and aligned FN microenvironment in the same fibroblast type, middle row *p*‐values compare pKO‐β1 and pKO‐αV/β1 fibroblasts in the same FN microenvironment, and bottom row *p*‐values compare pKO‐αV fibroblasts with other fibroblasts in the same FN microenvironment. *P* values were calculated using two‐tailed Mann–Whitney test. *P* values lower than 0.05 were considered significant. In (f), each truncated violin plot indicates F‐actin orientation distributions from different samples. The curves of grey violin plots cover only to the minimum and maximum values. The middle red lines indicate the median of the data sets, whereas the other two dotted lines indicate quartiles (25th and 75th percentiles). SD indicates standard deviation values. The number of biological replicates for the data in figure 1e–g is 8 for all conditions.

Electrospun random and aligned polycaprolactone (PCL) nanofibers coated with FN served as healthy and fibrotic FN models to investigate the effect of the fibrosis‐mimetic aligned nanofibrous FN microenvironment on fibroblasts expressing FN‐binding integrins (Figure 1a,b; Figure S1). We used PCL nanofibers due to their excellent biocompatibility, biomimicry, and biodegradation [22]. The diameters of the random and aligned nanofibers were 692.5 ± 105.4 nm (average ± SD, from 8 samples) and 705.0 ± 86.4 nm (average ± SD, from 8 samples) that are under the physiological ECM nanofiber diameter ranges (100–1000 nm) [23]. After coating, FN is fully and homogeneously distributed through both random and aligned nanofibers (Figure 1a; Figure S2). The estimated Young's Modulus of random (0.55 ± 0.17 MPa, mean ± SEM from *n* = 5) and aligned (0.90 ± 0.29 MPa, mean ± SEM from *n* = 5) nanofibers was not significantly different (Figure S3) suggesting that we can decouple the effect of stiffness from our experiments while testing the effect of FN alignments on cell responses. The configuration of random and aligned nanofibrous FN microenvironments is reproducibly controlled and significantly different (Figure 1b), suitable for testing the effect of the FN alignment on the molecular mechanisms of integrin‐mediated fibrogenesis. To investigate the unknown role of FN‐binding integrins in sensing the aligned FN microenvironment, we also utilized engineered pan‐integrin null (pKO) fibroblasts reconstituted with FN‐binding α5β1 integrins (pKO‐β1), αV‐class integrins (pKO‐αV), or both integrin classes (pKO‐αV/β1) [12].

We first hypothesized that the aligned FN microenvironment employs specific integrins to trigger early fibrotic cell fate decisions. To test this hypothesis, we cultured pKO‐αV, pKO‐β1, and pKO‐αV/β1 fibroblasts on random and aligned nanofibrous FN microenvironments and measured single‐cell behaviors to verify early fibrotic characteristics associated with the aligned nanofibrous FN microenvironment (Figure 1c). After 90 min of culture, cells were fixed and stained for filamentous actin (F‐actin) to analyze cell morphology, actin orientation, and paxillin to assess focal adhesion formation during early single‐cell growth (Figure 1d).

All pKO fibroblasts exhibited low coherency, or alignment, of cell morphology (Figure 1d,e; Figure S4) on the random FN microenvironment without any significant difference among FN‐binding integrin types. On the aligned FN microenvironments, pKO‐αV fibroblasts showed a similar coherency compared to those on the random FN microenvironment. Conversely, pKO‐β1 and pKO‐αV/β1 fibroblasts exhibited significantly higher coherencies on the aligned FN microenvironment than the random FN microenvironment. Coherency of pKO‐β1 fibroblasts is significantly greater than that of pKO‐αV (by 103%) and pKO‐αV/β1 (by 62%) fibroblasts on the aligned FN microenvironment. This finding indicates fibroblasts employ α5β1 integrins to sense the aligned nanofibrous cue of the FN microenvironment and accelerate cell elongation through the unidirectional axis. However, α5β1 integrin‐mediated FN alignment sensing is reduced when both αV‐class and α5β1 integrins co‐exist.

Next, the distributions of F‐action orientations (Figure 1d,f; Figure S4) of all pKO fibroblasts were largely spread on the random FN microenvironments showing the high standard deviation (SD) values (> 20°). On the contrary, all pKO fibroblasts showed significantly lower SD values (< 14°) on the aligned FN microenvironment indicating actin cytoskeleton within cells are rearranged to highly align them with the surrounding FN directional cues. Among pKO fibroblasts, pKO‐β1 fibroblasts showed the most highly consolidated distribution of F‐actin orientations with the lowest SD value (6.58%–60% lower than that of pKO‐αV fibroblasts in the same condition). These data suggest that α5β1 integrins sense the aligned FN microenvironment and facilitate the unidirectional actin filament arrangement that could contribute to generating contractile stress fibers and profibrotic activation in fibroblasts [24].

Focal adhesion area per cell was further analyzed by measuring paxillin‐positive staining area per cell (Figure 1d,g; Figure S4) [25]. In line with previous studies where adhesion initiation and maturation on 2D fibronectin coatings were strengthened via α5β1 integrins [12, 26, 27], focal adhesion area on the random FN microenvironment was significantly lower in pKO‐αV fibroblasts relative to pKO‐β1 and pKO‐αV/β1 fibroblasts. Focal adhesion area of pKO‐β1 and pKO‐αV/β1 fibroblasts on the random FN microenvironment was not significantly different. When cultured on the aligned FN microenvironment, focal adhesion areas of all pKO fibroblasts were significantly higher (> 73%) than those cultured on the random FN microenvironment. Focal adhesion area on the aligned FN microenvironment was significantly higher in pKO‐β1 fibroblasts relative to pKO‐αV (by 50%) and pKO‐αV/β1 (by 37%) fibroblasts, whereas pKO‐αV and pKO‐αV/β1 fibroblasts exhibited a similar focal adhesion. This data demonstrates that α5β1 integrins sense and respond to both random and aligned nanofibrous FN microenvironments. α5β1 integrin‐mediated focal adhesion is strengthened in sensing the aligned FN microenvironment. This effect is absent when both α5β1 and αV‐class integrins co‐exist, suggesting that mechanosensing the aligned nanofibrous FN is solely facilitated by α5β1 integrin.

Collectively, these results suggest that, while αV‐class integrins indicated limited mechanosensitive responses, α5β1 integrins sense and respond to the aligned FN microenvironment to facilitate cell elongation, F‐actin alignment, and focal adhesion formation during early cell growth. To date, understanding the crosstalk between FN‐ binding αV‐class and α5β1 integrins remains elusive and showed different results depending on the surrounding FN microenvironment, time and biological scales. For instance, during adhesion initiation within a few minutes of cell seeding, fibroblasts expressing both integrins showed significantly lower adhesion strengthening compared to fibroblasts expressing only α5β1 integrins [27]. Conversely, our recent study showed that fibroblasts expressing both integrins established similar adhesion forces to fibrous ECM matrices compared to fibroblasts expressing only α5β1 integrins [26]. Thereby, it is surprising to see the neutralizing action between αV‐class and α5β1 integrins on coherency and focal adhesion area of single fibroblasts in response to aligned FN microenvironment at 90 min.

### 2.2 The Aligned Nanofibrous FN Microenvironment Activates Early Profibrotic Actin Fiber Formation via α5β1 Integrins

During fibrosis, fibroblasts become profibrotic by increasing cell adhesion/migration and excessive ECM deposition [2]. One of the key profibrotic signals to trigger fibrosis is alpha‐smooth muscle actin (α‐SMA) [5]. Actin patch formation involving α‐SMA and F‐actin [28] is a critical parameter in fibrogenesis that triggers the transition of fibroblasts to contractile myofibroblasts [29]. Therefore, we hypothesize that the aligned FN microenvironment accelerates early profibrotic characteristics such as α‐SMA and stress fiber formation.

**FIGURE 2:**
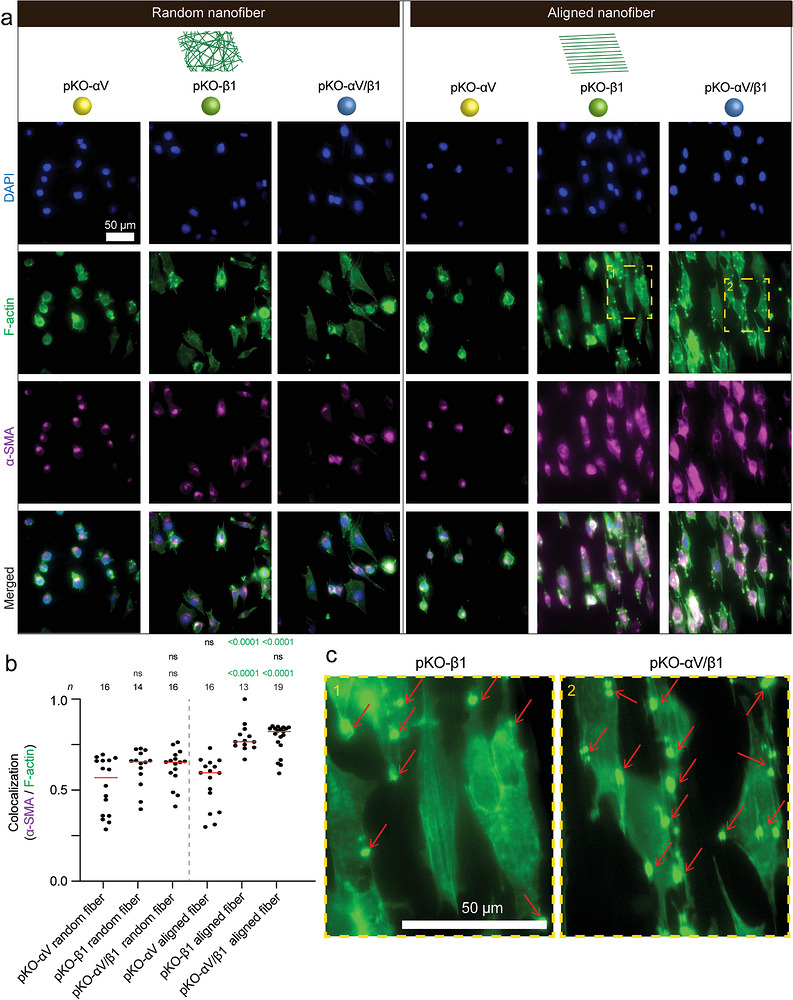
Early fibrotic marker (α‐SMA) expression analysis of pKO fibroblasts in response to the nanofibrous FN alignment. (a) Representative immunofluorescence images of pKO fibroblasts grown on the random and aligned FN microenvironments after 90 min of culture stained with DAPI (blue), F‐actin (green), and α‐SMA (magenta) with (b) α‐SMA/F‐actin colocalization analysis. (c) Zoom‐in F‐actin images of pKO‐β1 and pKO‐αV/β1 grown on the aligned FN microenvironment from the yellow dotted areas in (a) to show actin patch formation. In (b), dots represent co‐localization of individual samples and red bars represent the median. Top row *p*‐values compare random and aligned FN microenvironment in the same fibroblast type, middle row *p*‐values compare pKO‐β1 and pKO‐αV/β1 fibroblasts in the same FN microenvironment, and bottom row *p*‐values compare pKO‐αV fibroblasts with other fibroblasts in the same FN microenvironment. *P* values were calculated using two‐tailed Mann–Whitney test. *P* values lower than 0.05 were considered significant. The number of biological replicates for data in figure 2b is 4 for all conditions.

To test this hypothesis, we first measured co‐localization of α‐SMA and F‐actin signals (Figure 2a,b; Figure S5), where higher degrees of co‐localization represent greater profibrotic fibroblast activation [30]. On the random FN microenvironment, there was no difference between pKO‐αV, pKO‐β1, and pKO‐αV/β1 fibroblasts in the co‐localization of α‐SMA and F‐actin signals. On the other hand, co‐localization of α‐SMA and F‐actin signals on the aligned FN microenvironment significantly increased in pKO‐β1 (by 28%), and pKO‐αV/β1 (by 38%) fibroblasts relative to pKO‐αV fibroblasts, while there is no significant different between pKO‐β1 and pKO‐αV/β1 fibroblasts in this case. pKO‐β1, and pKO‐αV/β1 fibroblasts further exhibited significantly higher α‐SMA/F‐actin co‐localization on the aligned FN microenvironment compared to the random FN microenvironment, whereas pKO‐αV fibroblasts presented a similar colocalization ratio on both the random and aligned FN microenvironment. Moreover, notable F‐actin patch formation (Figure 2c) was only found in pKO‐β1, and pKO‐αV/β1 fibroblasts cultured on the aligned FN microenvironment, not any other conditions.

In summary, our results indicate that only α5β1 integrins trigger early profibrotic α‐SMA expression and F‐actin patch formation in response to the aligned FN microenvironment. There is no indication of synergistic crosstalk between α5β1 and αV‐class integrins for this alignment‐sensing process. A previous study showed higher expression of both α‐SMA and fibronectin in cancer‐associated fibroblasts indicating their clear correlation in fibrotic fibroblast phenotype [31]. Also, fibroblast adhesions enriched in α5β1 integrins have been reported to connect fibrous FN microenvironment to the contractile actin cytoskeleton [32]. This is in line with our observations where only α5β1 integrins in fibroblasts increase α‐SMA expression and F‐actin formation by the aligned FN microenvironment.

### 2.3 Early α5β1 Integrin Appearance in Single Fibroblast is Elevated by the Aligned Nanofibrous FN Microenvironment

**FIGURE 3:**
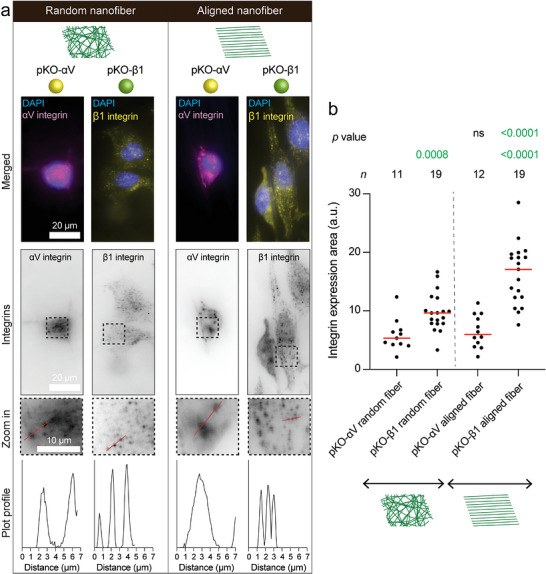
Early FN‐binding integrin appearance analysis of pKO fibroblasts in response to the nanofibrous FN alignment. (a) Representative immunofluorescence images of pKO‐αV or pKO‐β1 fibroblasts grown on the random and aligned FN microenvironments after 90 min of culture stained with DAPI (blue), αV integrins (magenta), or β1 integrins (yellow) in the merged image panel. In the integrin image panel, only αV integrins in pKO‐αV fibroblasts or β1 integrins in pKO‐β1 fibroblasts are showed with the expanded images in the Zoom in panel to clearly visualize the integrin clusters. Plot profiles were taken from the red lines in the Zoom in images to show the size of integrin clusters. (b) Integrin appearance area analysis. Dots represent individual samples and red bars the median. Top row *p*‐values compare random and aligned FN microenvironment in the same fibroblast type. Bottom row *p*‐values compare pKO‐αV and pKO‐β1 fibroblasts in the same FN microenvironment. *p* values were calculated using two‐tailed Mann–Whitney test. *p*‐values lower than 0.05 were considered significant. The number of biological replicates for data in figure 3b is 4 for all conditions.

Next, we aim to probe the appearance of α5β1 or αV‐class integrins in response to the FN alignment. To test this, we immunostained and imaged αV‐class or α5β1 integrins in pKO‐αV or pKO‐β1 fibroblasts cultured for 90 min on the random and aligned FN microenvironments, respectively (Figure 3; Figure S6).

Similar to the previous studies performed on 2D ECM coatings [12], on both random and aligned FN microenvironments, larger focal adhesion (> 2 µm) were observed in pKO‐αV fibroblasts compared to smaller nascent adhesion (< 2 µm) in pKO‐β1 fibroblasts on both the random and aligned FN microenvironments (Figure 3a). αV‐class integrin expression per single pKO‐αV fibroblast was similar on both the random and aligned FN microenvironments (Figure 3b). However, α5β1 integrin expression per single pKO‐β1 fibroblast was significantly elevated in the aligned FN microenvironment (by 78%) compared to the random FN microenvironment. These findings support that α5β1 integrins play a crucial role in protrusive nascent adhesion formation [12] that is reinforced by the alignment of the nanofibrous FN microenvironment.

Altogether, our single‐cell analyses (Figures 1, 2, 3) indicate that, during early adhesion establishment, fibroblasts especially employ α5β1 integrins to respond to the aligned FN microenvironment to expedite cell/actin alignment, nascent focal adhesion formation, α‐SMA expression, and F‐actin patch development. This demonstrates that the α5β1 integrin‐mediated sensing of the nanofibrous FN alignment rapidly drives protrusive and profibrotic fibroblast fates at the onset of adhesion.

### 2.4 The Aligned Nanofibrous FN Microenvironment Facilitates α5β1 Integrin‐Mediated Fibrotic Tissue Deposition

**FIGURE 4:**
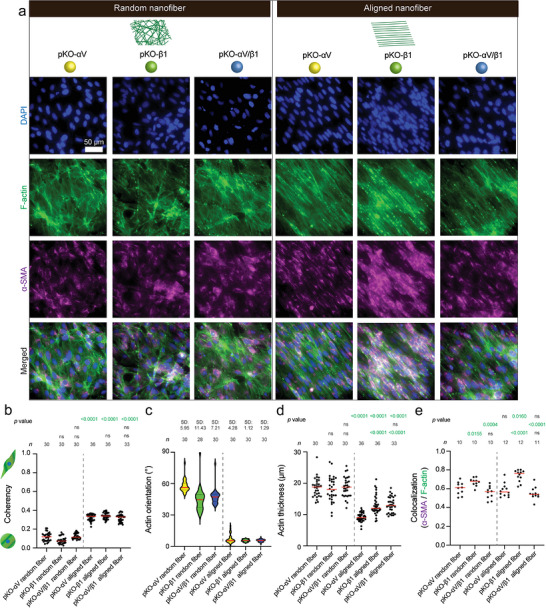
Fibrotic tissue development by fibroblasts in response to the nanofibrous FN alignment. (a) Representative immunofluorescence images of pKO fibroblasts grown on the random and aligned FN microenvironments after 3 days of culture stained with DAPI (blue), F‐actin (green), and α‐SMA (magenta) with (b) cell coherency (or alignment), (c) F‐actin orientation, (d) F‐actin thickness, and e) α‐SMA/F‐actin colocalization analysis. In (b), (d), and (e), dots represent data sets from individual samples and red bars the median. Top row *p*‐values compare random and aligned FN microenvironment in the same fibroblast type, middle row *p*‐values compare pKO‐β1 and pKO‐αV/β1 fibroblasts in the same FN microenvironment, and bottom row *p*‐values compare pKO‐αV fibroblasts with other fibroblasts in the same FN microenvironment. *P* values were calculated using two‐tailed Mann–Whitney test. *p* values lower than 0.05 were considered significant. In (c), each truncated violin plot indicates F‐actin orientation distributions from different samples. The curves of grey violin plots cover only to the minimum and maximum values. The middle red lines indicate the median of the data sets, whereas other two dotted lines indicate quartiles (25th and 75th percentiles). SD indicates standard deviation values. The number of biological replicates for data in figure 4b–e is 3 for all conditions.

To determine whether aligned microenvironment sensing continues to promote fibrotic tissue formation at later stages of the cellular life cycle, we extended our study to assess the role of FN alignment and FN‐binding integrins in fibrotic tissue formation over 3 days (Figure 4a).

We first analyzed tissue alignment to assess if fibroblasts develop random (healthy) or aligned (fibrotic) tissues (Figure 4b; Figure S7). All pKO fibroblasts formed random tissues on the random FN microenvironment and aligned tissues on the aligned FN microenvironment, respectively, regardless of expressing integrin types. Similarly, all pKO fibroblasts exhibited randomly distributed F‐actin orientations on the random FN microenvironment, whereas they showed highly unidirectional F‐actin orientations on the aligned FN microenvironment (Figure 4c). Among pKO fibroblasts, pKO‐β1 fibroblasts on the aligned FN microenvironment presented the least deviation of F‐actin orientation values. Furthermore, there was no difference in actin thickness in all pKO fibroblasts cultured on the random FN microenvironment (Figure 4d; Figure S7). On the aligned FN microenvironment, actin thickness was significantly increased in pKO‐β1 (by 34%) and pKO‐αVβ1 (by 43%) fibroblasts compared to pKO‐αV fibroblasts, while there was no difference on actin thickness between pKO‐β1 and pKO‐αVβ1 fibroblasts. Next, we probed α‐SMA expression in tissues, which is one of the major fibrosis markers (Figure 4e; Figure S7). Colocalization of α‐SMA and F‐actin was significantly higher in pKO‐β1 fibroblasts relative to pKO‐αV and pKO‐αVβ1 fibroblasts regardless of FN alignment. However, pKO‐β1 fibroblasts considerably elevated colocalization of α‐SMA and F‐actin on the aligned FN microenvironment (by 12%) compared to the random FN microenvironment, whereas pKO‐αV and pKO‐αVβ1 fibroblasts presented similar values regardless of FN alignment.

These results explain that α5β1 integrins strengthen tissue alignment, thickening, and α‐SMA positive tissue formation, which are all major characteristics of fibrotic tissues [33], in response to the aligned FN microenvironment. Previously, 2D FN coatings have been shown to promote nuclear elongation and tube formation of myofibroblasts in vitro compared to gelatine and laminin [34]. On top of this, our study further suggests that alignment as well as FN composition facilitates tissue alignment and thickening similar to fibrotic tissues.

### 2.5 Aligned Fibrotic Fibronectin and Collagen I Deposition are Boosted via α5β1 Integrins in Response to the Aligned Nanofibrous FN

**FIGURE 5:**
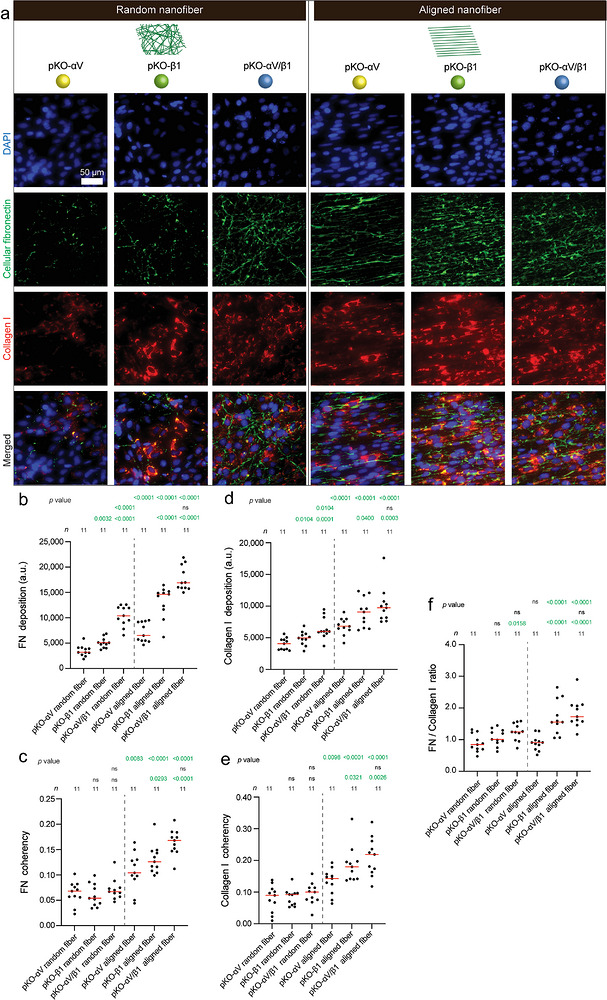
Fibrotic ECM deposition by fibroblasts in response to the nanofibrous FN alignment. (a) Representative immunofluorescence images of pKO fibroblasts grown on the random and aligned FN microenvironments after 3 days of culture stained with DAPI (blue), cellular FN (green), and collagen I (red) with (b) FN deposition, (c) FN coherency (or alignment), (d) collagen I deposition, e) collagen I coherency (or alignment), and f) cellular FN /collagen I deposition ratio analysis. In (b–f), dots represent data sets from individual samples and red bars the median. Top row *p*‐values compare random and aligned FN microenvironment in the same fibroblast type, middle row *p*‐values compare pKO‐β1 and pKO‐αV/β1 fibroblasts in the same FN microenvironment, and bottom row *p*‐values compare pKO‐αV fibroblasts with other fibroblasts in the same FN microenvironment. *P* values were calculated using two‐tailed Mann–Whitney test. *p* values lower than 0.05 were considered significant. The number of biological replicates for data in figure 5b–f is 3 for all conditions.

To investigate how cells employ integrins to modulate FN and collagen deposition in response to FN alignment, we evaluated the amount, orientation, and ratio of cellular FN and collagen I deposition after culture of pKO‐αV, pKO‐β1, and pKO‐αVβ1 fibroblasts for 3 days on random and aligned FN microenvironments (Figure 5a).

Cellular FN deposition on the random FN microenvironment was increased on pKO‐β1 (by 59%) and pKO‐αVβ1 (by 225%) fibroblasts relative to pKO‐αV fibroblasts (Figure 5b; Figure S8). Compared to the random FN microenvironment, all pKO fibroblasts exhibited significantly greater cellular FN deposition on the aligned FN microenvironment. On the aligned FN microenvironment, the cellular FN deposition was significantly decreased in pKO‐αV fibroblasts relative to pKO‐β1 (by 133%) and pKO‐αVβ1 (by 159%) fibroblasts while pKO‐β1 and pKO‐αVβ1 fibroblasts showed no significant difference in the FN deposition. Next, the alignment (or coherency) of the deposited cellular FN was significantly higher on the aligned FN microenvironment compared to the random FN microenvironment regardless of cell type (Figure 5c; Figure S8). On the random FN microenvironment, there was no difference in the cellular FN alignment between pKO‐αV, pKO‐β1, and pKO‐αVβ1 fibroblasts. On the other hand, the cellular FN alignment was elevated in pKO‐β1 (by 38%) and pKO‐αVβ1 (by 62%) fibroblasts compared to pKO‐αV fibroblasts. pKO‐β1 and pKO‐αVβ1 fibroblasts exhibited a similar cellular FN orientation.

Similarly, collagen I deposition was significantly greater on the aligned FN microenvironment relative to the random FN microenvironment for all pKO fibroblasts (Figure 5d; Figure S8). On the random FN microenvironment, pKO‐αV fibroblasts exhibited significantly lower collagen I deposition compared to pKO‐β1 (by 22%) and pKO‐αVβ1 (by 47%) fibroblasts, while collagen I deposition was also higher on pKO‐αVβ1 fibroblasts (by 21%) relative to pKO‐β1 fibroblasts. On the aligned FN microenvironment, collagen I deposition was significantly increased on pKO‐β1 (by 33%) and pKO‐αVβ1 (by 43%) fibroblasts relative to pKO‐αV fibroblasts. There was no difference in collagen I deposition between pKO‐β1 and pKO‐αVβ1 fibroblasts cultured on the aligned FN microenvironment. The alignment (or coherency) of the deposited collagen I was significantly higher on the aligned FN microenvironment compared to the random FN microenvironment regardless of cell types (Figure 5e; Figure S8). All pKO fibroblasts showed similar collagen I orientation on the random FN microenvironment. The deposited collagen I alignment was elevated in pKO‐β1 (by 26%) and pKO‐αVβ1 (by 53%) fibroblasts compared to pKO‐αV fibroblasts while pKO‐β1 and pKO‐αVβ1 fibroblasts exhibited a similar collagen I alignment.

Lastly, cellular FN over collagen I deposition ratio was significantly elevated in pKO‐αVβ1 fibroblasts relative to pKO‐αV and pKO‐β1 fibroblasts on the random FN microenvironment (Figure 5f; Figure S8). On the aligned FN microenvironment, pKO‐β1 and pKO‐αVβ1 fibroblasts exhibited a significantly greater cellular FN to collagen I deposition ratio compared to pKO‐αV fibroblasts. There was no difference between pKO‐β1 and pKO‐αVβ1 fibroblasts. FN/collagen I deposition in pKO‐β1 and pKO‐αVβ1 fibroblasts significantly increased on the aligned FN microenvironment compared to the random FN microenvironment, whereas pKO‐αV fibroblasts showed similar FN/collagen I deposition under both conditions.

In summary, these ECM deposition results suggest that fibroblasts use α5β1 integrins to activate aligned cellular FN and collagen I deposition. The cellular FN deposition scale was larger than collagen I deposition scale at this time point. In previous 2D FN studies, fibronectin has been shown to play a crucial role in organizing other ECM proteins such as collagen I in the tissues and modulating cell responses in a mechanical force‐dependent manner [35, 36]. This supports our observations where the aligned FN microenvironment promotes deposition of both cellular FN and collagen I to form fibrotic tissues.

### 2.6 The Aligned Nanofibrous FN Microenvironment Reinforces α5β1 Integrin Appearance in Fibrotic Tissues

**FIGURE 6:**
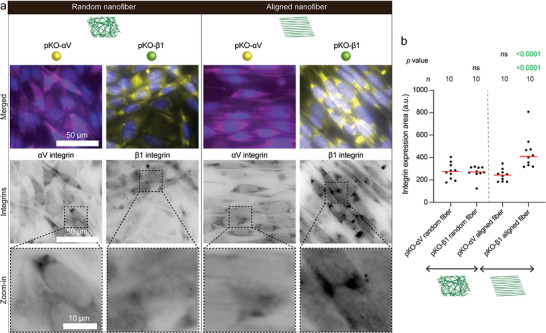
FN‐binding integrin appearance analysis of tissues formed by pKO fibroblasts in response to the nanofibrous FN alignment. a) Representative immunofluorescence images of pKO‐αV or pKO‐β1 fibroblasts grown on the random and aligned FN microenvironments after 3 days of culture stained with DAPI (blue), αV integrins (magenta), or β1 integrins (yellow) in the merged image panel. In the integrin image panel, only αV integrins in pKO‐αV fibroblasts or β1 integrins in pKO‐β1 fibroblasts are shown with the expanded images in the Zoom in panel to clearly visualize the integrin clusters. b) Integrin appearance area analysis. Dots represent individual samples and red bars the median. Top row *p*‐values compare random and aligned FN microenvironment in the same fibroblast type. Bottom row *p*‐values compare pKO‐αV and pKO‐β1 fibroblasts in the same FN microenvironment. *p*‐values were calculated using two‐tailed Mann–Whitney test. *P* values lower than 0.05 were considered significant. The number of biological replicates for the data in figure 6b is 3 for all conditions.

To assess the distinctive role of αV‐class and α5β1 integrins in fibrotic tissue formation, we measured the appearance of αV‐class and α5β1 integrins in tissues produced by pKO‐αV and pKO‐β1 fibroblasts after 3 days of culture (Figure 6a). Compared to the single cell data in Figure 3, αV‐class and α5β1 integrin appearance are considerably increased (over 20‐fold) in the tissue data (Figure 6b; Figure S9). On the random FN microenvironment, αV‐class integrin appearance was similar to α5β1 integrin expression. Conversely, α5β1 integrin appearance was significantly elevated by 68% relative to αV‐class integrin appearance on the aligned FN microenvironment. There was no significant difference in αV‐class integrin appearance between the random and aligned FN microenvironment, whereas α5β1 integrin appearance significantly increased in the aligned FN microenvironment by 51% compared with the random FN microenvironment. We also found that pKO‐β1 fibroblasts grown on the aligned FN microenvironment developed large clusters of α5β1 integrins at the edges of cells in the fibrotic tissue, which indicates strong cell‐ECM and cell‐cell adhesions, a hallmark of fibrosis [37, 38]. This is in line with a previous study, which showed α5β1 integrins together with αVβ5 integrins are involved in fibrotic ECM deposition and fibroblasts‐to‐myofibroblasts activation in pancreatic ductal adenocarcinoma‐associated cell lines [39].

## 3 Discussion and Conclusion

FN is one of the major structural ECM proteins that form complex and heterogeneous nanofibrous networks [6]. Over their lifespan, cells employ FN‐binding integrins (αV‐class and α5β1 integrins) to sense and respond to the surrounding FN cues [10, 26, 40]. During fibrosis, FN overproduction drives excessive ECM deposition by activating collagen overproduction and tissue stiffening [7, 8, 9, 41]. So far, it has been established that αV‐class integrins enhance cytoskeletal contractility, pro‐fibrotic fibroblast activation, and ECM deposition/stiffening [42, 43], while β1 integrins also facilitate pro‐fibrotic fibroblast activation, actin contractility, and ECM production [44]. These previous studies confirm the crucial role of both αV‐class and α5β1 integrins during fibrosis [45], but do not clarify whether and how αV‐class and α5β1 integrins mechanosense FN cues (e.g., fibrous structure/alignment) and modulate fibrogenesis at different time points.

To further obtain molecular insight into the FN‐binding integrin‐mediated mechanotransduction, recent studies have delved into FN‐binding integrin‐mediated mechanisms in vitro. For instance, α5β1 integrins engaged with 2D FN coatings strengthen fibroblast adhesion initiation in less than 1 s with a biphasic behavior [27], while αV‐class integrins help α5β1 integrins to cluster and strengthen adhesion initiation [46]. α5β1 integrins in fibroblasts play a crucial role in fibrillogenesis of cell‐secreted FN on 2D ECM coating after 1 h of cell culture [47]. Beyond the conventional and non‐biomimetic 2D substrates, our recent study found that, compared to 2D FN coating, fibrillar fibronectin matrices employ not αV‐class integrins, but α5β1 integrins to facilitate adhesion initiation, migration, and tissue formation throughout their lifespan [26, 40]. Although these studies have revealed how FN‐binding integrins respond to the engineered FN substrates over time, it remains unknown yet how fibroblasts employ particular FN‐binding integrins in response to the alignment of biomimetic nanofibrous FN microenvironment and how such fibrous FN alignment sensing influences pro‐fibrotic cell responses and fibrotic tissue development.

**FIGURE 7:**
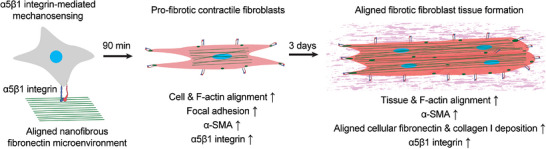
Schematic illustration of FN‐binding α5β1 integrin‐mediated mechanosensing of aligned nanofibrous FN microenvironment to activate early pro‐fibrotic contractile fibroblast responses and continuously fibrotic tissue formation.

In this manuscript, we, for the first time, investigate the role of the nanofibrous FN alignment on modulating FN‐binding integrins and pro‐fibrotic fibroblast behavior at different time points (Figure 7). Compared to the current 2D models, this model better represents the nanofibrous microstructure and alignment of healthy and fibrotic human tissues. The 90 min time point selected is a standard time point for immunostaining analysis of a single cell response [12, 46]. Of focal adhesion proteins, we chose paxillin for our focal adhesion analysis, because paxillin localizes to focal adhesions and interconnects integrin‐mediated ECM adhesion [12] by recruiting intracellular molecules such as FAK, Src, vinculin, and actin cytoskeleton [48]. Investigating and quantifying paxillin provide new insights into how FN‐binding integrins regulate fibroblast focal adhesion in response to the aligned FN microenvironment. To further characterize a tissue formation response and test whether the alignment sensing identified from the single cell response at 90 min continues at later stage, we selected a time point (3 days) at which fibroblasts with a seeding density of 100 000 cells/sample consistently start to aggregate and form a tissue. This timescale likewise mimics fibroblast aggregation and clustering during tissue regeneration [49].

For all experiments, we believe that pKO‐αV/β1 fibroblasts should be considered as baseline for integrin‐mediated effects in sensing FN alignment. This is based on a previous study where there is no difference between pKO‐αV/β1 and parental pKO fibroblasts in adhesion initiation and strengthening [27]. Therefore, we speculate that there would be no significance difference between pKO‐αV/β1 and parental pKO fibroblasts since FN‐mediated adhesion and integrin‐mediated effects mainly come from α5β1 and αV class integrins which pKO‐αV/β1 fibroblasts fully express. To assure normal cell growth and minimize exogenous proteins from FBS in the cell culture medium, we also used 1% FBS low serum condition compared to typical 10% FBS in our experiments. It should be noted that there is a potential exogenous FN concentration from 1% FBS although its amount is expected to be considerably low compared to immobilized FN coating concentration.

We first found that, during the initial cell adhesion at 90 min, fibroblasts employ not αV‐class integrins, but α5β1 integrins to further strengthen cell and F‐actin alignment, focal adhesion area, and α‐SMA expression. This is in line with previous studies where aligned cells and F‐actin create high contractile forces that is associated with α‐SMA activation, a hallmark of pro‐fibrotic fibroblast differentiation [28, 37]. The escalated pro‐fibrotic fibroblast behavior elicits greater expressions of small nascent adhesions by α5β1 integrins in single fibroblasts that indicate its protrusive nature toward becoming cancerous cell types [12, 50].

Subsequently, we verify whether, in response to the nanofibrous FN alignment, α5β1 integrin‐mediated early pro‐fibrotic cell fate decisions continue or change to produce fibrotic tissues at a later stage of cell lifespan. Interestingly, after 3 days of culture, fibroblasts continue to employ only α5β1 integrins to respond to the aligned FN microenvironment by forming aligned, thick, and α‐SMA positive tissues that match with the major characteristics of native fibrotic tissues [51, 52]. Furthermore, the aligned FN microenvironment activates deposition of aligned cellular fibronectin [41, 53, 54] and collagen I [19, 20] matrices via α5β1 integrins in fibrotic tissues that are the hallmark of fibrosis in terms of ECM remodeling. Generally, we did not observe any consistent or obvious synergistic effect between α5β1 and αV class integrins when they both express. But it was rather α5β1‐mediated effect compared to αV class integrins. This is in line with our recent study reporting that fibroblasts expressing both integrins established similar adhesion forces to fibrous ECM matrices compared to fibroblasts expressing only α5β1 integrins without any synergistic effect among these two FN‐binding integrins [26].

In conclusion, our findings define the role of FN‐binding α5β1 integrins to mechanosense the alignment of nanofibrous FN microenvironment and trigger early pro‐fibrotic cell fates and later fibrotic ECM/tissue production. Considering the understudied roles of FN alignments and FN‐binding integrins in mechanobiology and fibrosis [55], this study suggests that α5β1 integrins are an interesting potential target for treating fibrosis. It should be noted that we only probe the effect of nanofiber alignment in FN‐mediated mechanobiology in this study, and do not yet account for other aspects of mechanobiology such as stiffness. Likewise, which α5β1 integrin‐mediated intracellular proteins are involved in FN alignment‐dependent early pro‐fibrotic cell responses and later fibrotic tissue deposition and whether these are cell type‐specific remains to be investigated. While there is a clear effect of the nanofiber alignment on profibrotic fibroblast activation in our experiments, it should be noted that the stiffness of the random nanofibers is in the high kPa stiffness range close to stiffnesses of fibrotic tissues in general. Also, intracellular singling mechanisms in response to the ECM microarchitecture and ECM/integrin compositions together with broader gene expressions should be explored to provide thorough downstream molecular mechanisms.

## 4 Experimental Section/Methods

### 4.1 Engineering Random and Aligned Nanofibrous FN Microenvironment

Electrospun random and aligned polycaprolactone (PCL) nanofibers prepared on 24‐well plates (Merck) were commercially purchased and then coated with 50 µg/mL of human FN solution (from human plasma, Sigma‐Aldrich) in phosphate‐buffered saline (PBS, pH 7.4, Fisher Scientific Ireland) for 2 h at 37°C. Briefly, a polymer precursor solution including PCL is prepared in a highly volatile solvent such as 1,1,1,3,3,3,‐Hexafluoro‐2‐propanol or acetone at a concentration ranging from 1–50 wt% [56]. Then, a droplet of the precursor polymer solution will be loaded at a flow rate (0 – 50 mL/h) with a high voltage (0 – 30 kV) toward the electrode where the nanofibers are collected [56]. Depending on the collector system, the collected nanofibers can be randomly oriented or highly aligned [56]. After incubation, samples were washed three times with PBS before use. To verify FN coating with nanofiber samples, nanofibers coated with FN were incubated with amine‐reactive dye (Alexa Fluor 488 TFP ester, Fisher Scientific Ireland) for 30 min, washed three times with PBS, and imaged by epifluorescence microscopy (Olympus IX83). Additionally, FN‐coated nanofibers were also incubated with FN antibody (1:200, rabbit ployclonal, abcam, ab2413) and CF 555 and Goat Anti‐Rabbit IgG (H+L), CF 647, Sigma, SAB4600066 and SAB4600184) for 1 h each, washed three times with PBS, and imaged by the same microscopy. Prior to cell seeding, FN‐coated nanofibers were incubated with BSA (5% w/v) to block non‐specific protein adsorption.

### 4.2 Characterizing Random and Aligned Nanofibrous FN Microenvironment

The diameter of the random and aligned nanofibers was measured by using their bright field images taken by an epifluorescence microscopy (Olympus IX83). To assess the mechanical properties of the nanofibrous FN microenvironment, circular samples were cut from random and aligned nanofiber well plates used for our cell experiments. On a TA Discovery HR‐2 Rheometer, 8 mm geometry was lowered until the recorded axial force was 0.03. Viscoelastic properties were measured using a time sweep for 300 s at 0.1% strain and 1 Hz, and a frequency sweep from 0.1 to 100 Hz at 0.1% strain for *n* = 5 samples. Frequency sweep data and rubber elastic theory were used to convert the storage modulus to the elastic modulus, where E = 2G(1+ν), assuming a poison's ratio of 0.5 for bulk polymer networks [57, 58].

### 4.3 Fibroblast Cell Line Culture

pKO‐αV/β1, pKO‐β1, and pKO‐αV fibroblasts were provided and identified by R. Fässler [12]. Briefly, mice carrying null mutations for integrin genes were intercrossed, and fibroblasts were isolated and immortalized [12]. All integrins from the parental fibroblast clones (pan‐knockouts, pKO) were removed [12]. Then, αv or β1 or both complementary DNAs were reconstructed with the parental fibroblasts, resulting in cells expressing αv (pKO‐αV), β1 (, pKO‐β1) or αv and β1 (pKO‐αV/β1) integrins [12].

pKO‐αV/β1, pKO‐β1, and pKO‐αV fibroblasts were cultured on FN‐coated tissue culture flasks (VWR) with DMEM GlutaMAX (Fisher Scientific Ireland) containing fetal bovine serum (FBS, 10% w/v Sigma–Aldrich), penicillin (100 U mL^−1^), and streptomycin (100 µg mL^−1^, both Fisher Scientific Ireland).

### 4.4 Characterization of Single Fibroblast Responses

Once fibroblasts are ∼ 80% confluent, fibroblasts were washed with PBS followed by incubation with trypsin/EDTA (0.25% w/v, Sigma) for 2 min. Detached fibroblasts were seeded and allowed to attach to the random and aligned FN microenvironments in DMEM with FBS (1% w/v). After 90 min of culture, cells were washed three times with PBS and fixed by 4% v/v paraformaldehyde (PFA) with Triton X‐100 (0.05% v/v, Sigma) at room temperature for 10 min. After fixation, samples were washed three times with PBS and immunostained/imaged accordingly.

### 4.5 Characterization of Fibroblast Tissue Formation

Once fibroblasts were ∼ 80% confluent, they were washed with PBS followed by incubation with trypsin/EDTA (0.25% w/v, Sigma) for 2 min. Detached fibroblasts were seeded at a concentration of 100 000 cells/sample and allowed to attach to the random and aligned FN microenvironments in DMEM with FBS (1% w/v) for 3 days. To assure normal cell growth and minimize exogenous proteins from FBS in the cell culture medium, we used 1% FBS, low serum condition compared to typical 10% FBS used for cell passaging, in our experiments. Cell culture medium was exchanged every 2 days. After 3 days of culture, cells were washed three times with PBS and fixed by 4% v/v paraformaldehyde (PFA) with Triton X‐100 (0.05% v/v, Sigma) at room temperature for 10 min. After fixation, samples were washed three times with PBS and immunostained/imaged accordingly.

### 4.6 Immunostaining and Imaging

All antibodies were prepared in BSA (5% w/v) to block non‐specific binding. Fixed cells were incubated with paxillin (1:500, mouse monoclonal, Fisher Scientific Ireland, MA5‐31562), α‐SMA (1:500, rabbit polyclonal, Fisher Scientific Ireland, 16896595), αV integrin (1:500, rabbit polyclonal, Fisher Scientific Ireland, MA5‐32195), β1 integrin (1:500, mouse monoclonal, Fisher Scientific Ireland, 14‐0299‐82), cellular FN (1:200, mouse monoclonal, Sigma, SAB4200784), collagen I (1:200, rabbit polyclonal, Fisher Scientific Ireland, PA5‐95137) for 2 h at room temperature. After incubation, samples were washed three times with PBS and followed by secondary antibody incubation (Goat Anti‐Mouse IgG (H+L), CF 555 and Goat Anti‐Rabbit IgG (H+L), CF 647, Sigma, SAB4600066 and SAB4600184) together with DAPI (Fisher Scientific Ireland, 62248) or phalloidin (Phalloidin CF488A Conjugate, Biotium, 00042‐T) for 1 h at room temperature. After incubation, samples were washed three times with PBS and imaged by an epifluorescence microscopy (Olympus IX83).

### 4.7 Image Analysis

Nanofiber, cell, and F‐actin coherency and angles were calculated by using an ImageJ plug‐in (Orientation J) [59]. Briefly, the coherency (or alignment) ranges from 0 to 1. When the coherency approaches 1, it represents a preferential orientation of the fibers (e.g., aligned orientation). On the other hand, coherency values approaching 0 indicates there is no preferential orientation of the fibers (e.g., random orientation) [59]. Focal adhesion area, α‐SMA expression, F‐actin expression, integrin expression analysis was performed by using Image J to measure each antibody stained positive areas followed by the established protocol [25].

### 4.8 Statistical Analysis

We used unpaired, nonparametric two‐tailed Mann‐Whitney t‐tests to assess *p*‐values and statistical significance for comparing two separate, independent groups in each conditions considering potential heterogeneity of cell behaviors. *p* values lower than 0.05 were considered as significant. Prism (GraphPad Software) was used to perform all the statistical analysis.

## Funding

The City of Dublin Skin and Cancer Hospital Charity, University College Dublin.

## Conflicts of Interest

The authors declare no conflicts of interest.

## Supporting information




**Supporting File**: advs74984‐sup‐0001‐SuppMat.docx.

## Data Availability

The data that support the findings of this study are available from the corresponding author upon reasonable request.
