# Supplementary material for: Aligned Fibronectin Microenvironment Temporally Facilitates Profibrotic Fibroblast Activation via Integrin α5β1
Source: Adv Sci (Weinh). 2026 Mar 24;13(34):e00047. doi: 10.1002/advs.202600047 (PMC13285177; doi:10.1002/advs.202600047)
Supplement: Supplementary file 1 — Supporting File: advs74984‐sup‐0001‐SuppMat.docx. [file ADVS-13-e00047-s001.docx]

Supporting Information

Aligned Fibronectin Microenvironment Temporally Facilitates Profibrotic Fibroblast Activation *via* Integrin α5β1

Doğuhan Beyatli, Mika Brown, George-Radu Romanescu, and Seungkuk Ahn*


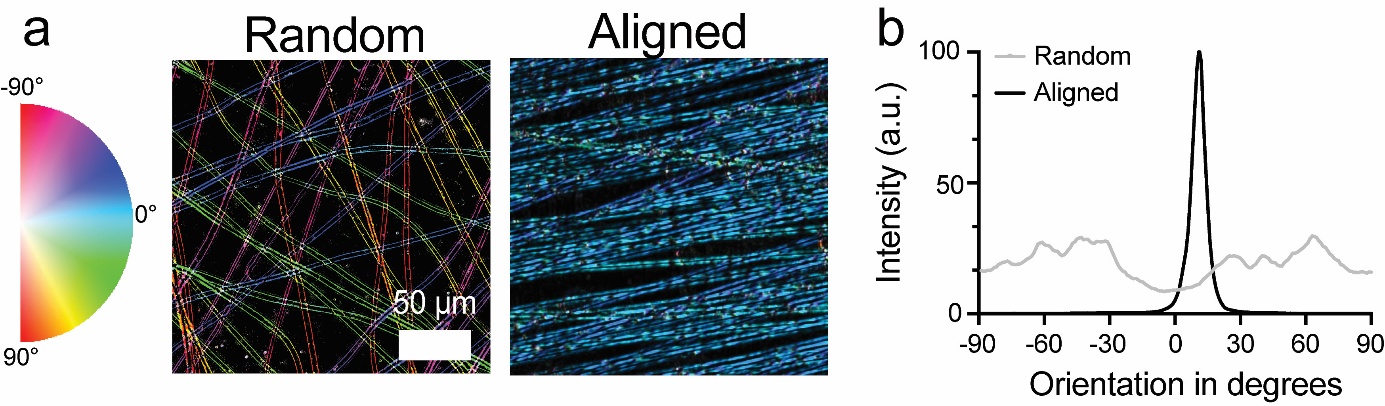


**Figure S1.** Random and aligned nanofiber orientation analysis with a) color-coded fluorescence images and b) intensity plot generated by Orientation J in ImageJ.


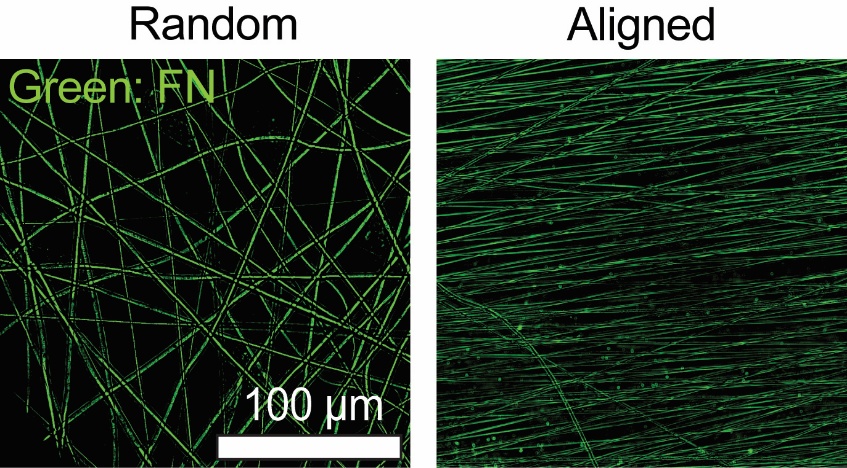


**Figure S2.** Random and aligned nanofibers stained with anti-FN antibody (green) to show the presence of FN on nanofibers.


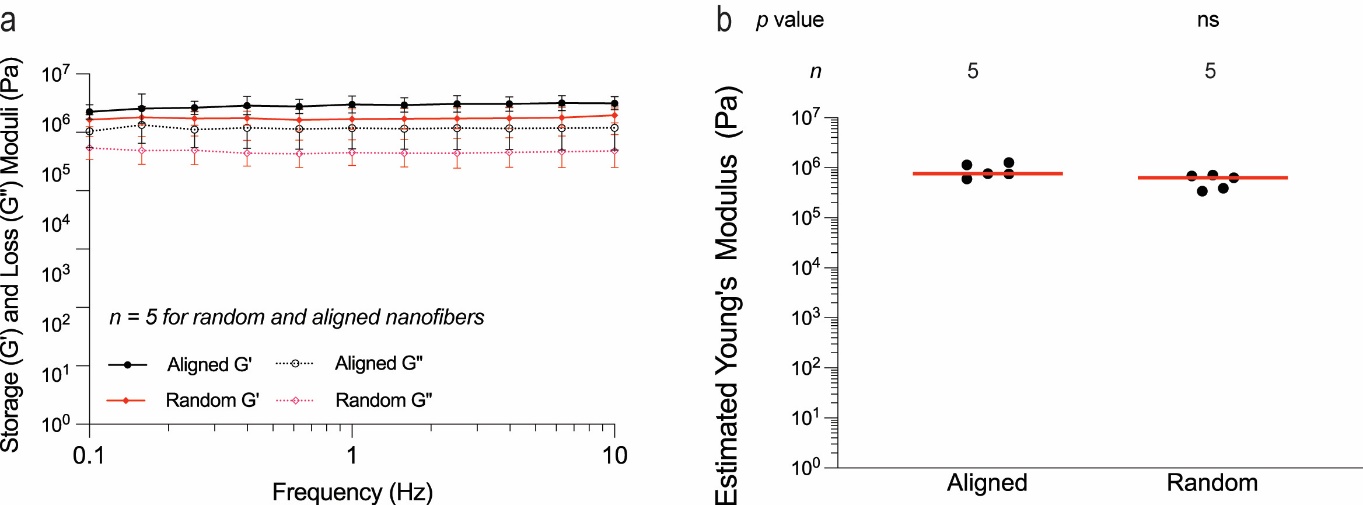


**Figure S3.** Rheology of random and aligned nanofibers to investigate their modulus. a) Frequency sweep curves of random and aligned nanofibers with G’ and G” values. b) Estimated Young’s Modulus calculated from the frequency sweep data with rubber elastic theory, showing no significant difference in Young’s Modulus between random and aligned nanofibers (*p* value: 0.056, not significant). *P* values were calculated using two-tailed Mann–Whitney test. The number of biological replicates for data is 5 for all conditions.


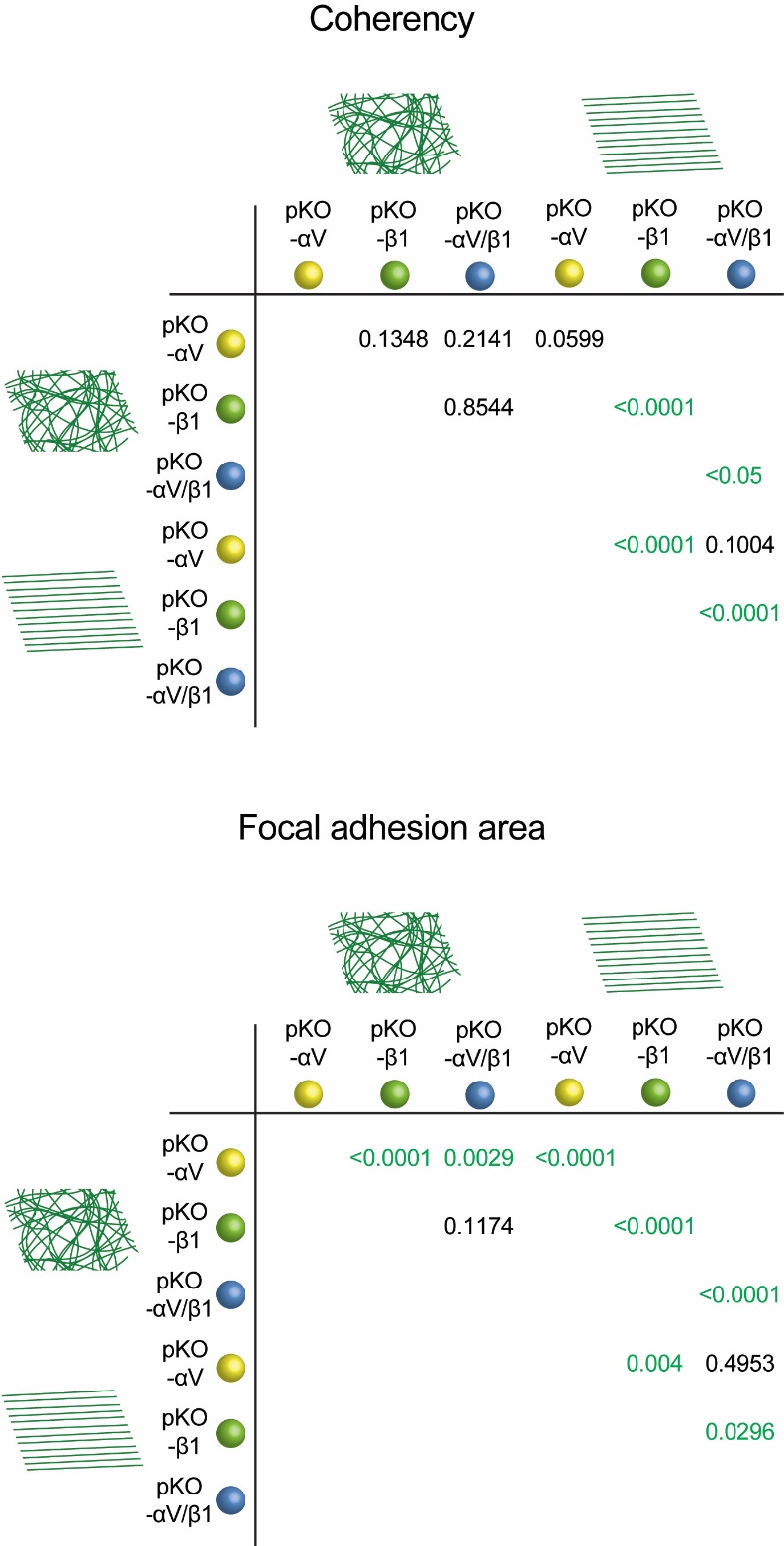


**Figure S4.** Statistical analysis of fibroblast coherency and focal adhesion area data in Figure 1e and 1g. *P* values were calculated using the two-tailed Mann-Whitney test. *P* values lower

than 0.05 (green) are considered significant.


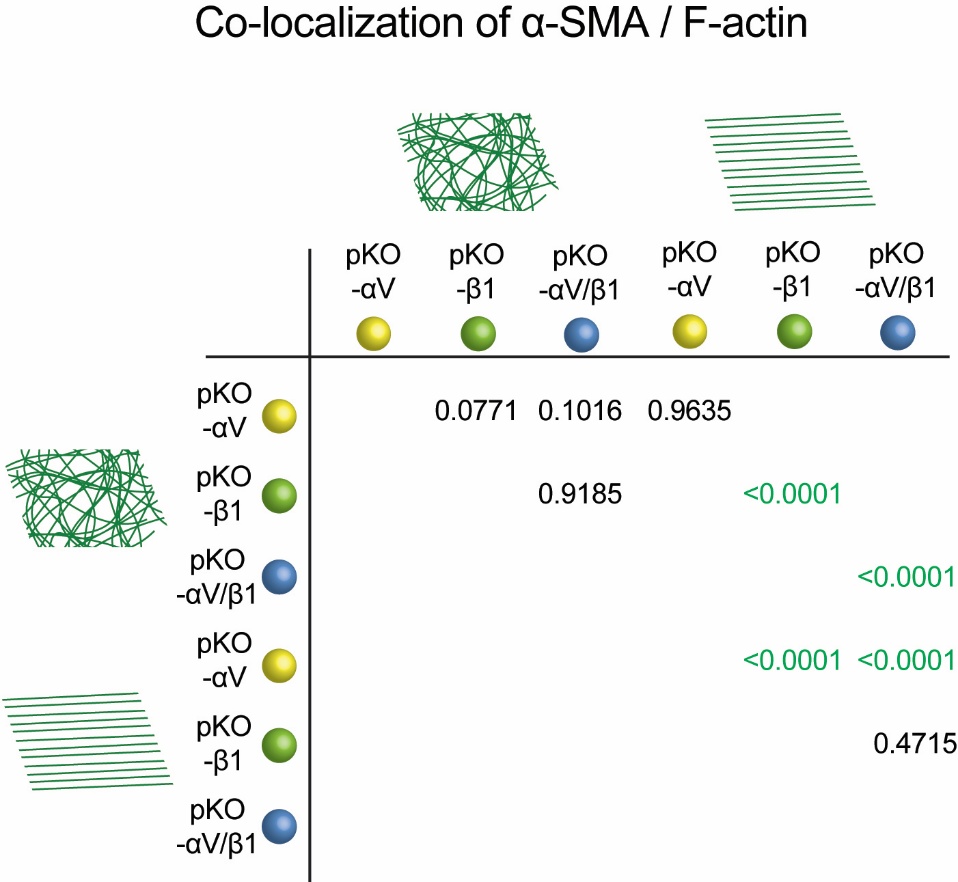


**Figure S5.** Statistical analysis of α-SMA/F-actin co-localization data in Figure 2b. *P* values were calculated using the two-tailed Mann-Whitney test. *P* values lower than 0.05 (green) are considered significant.


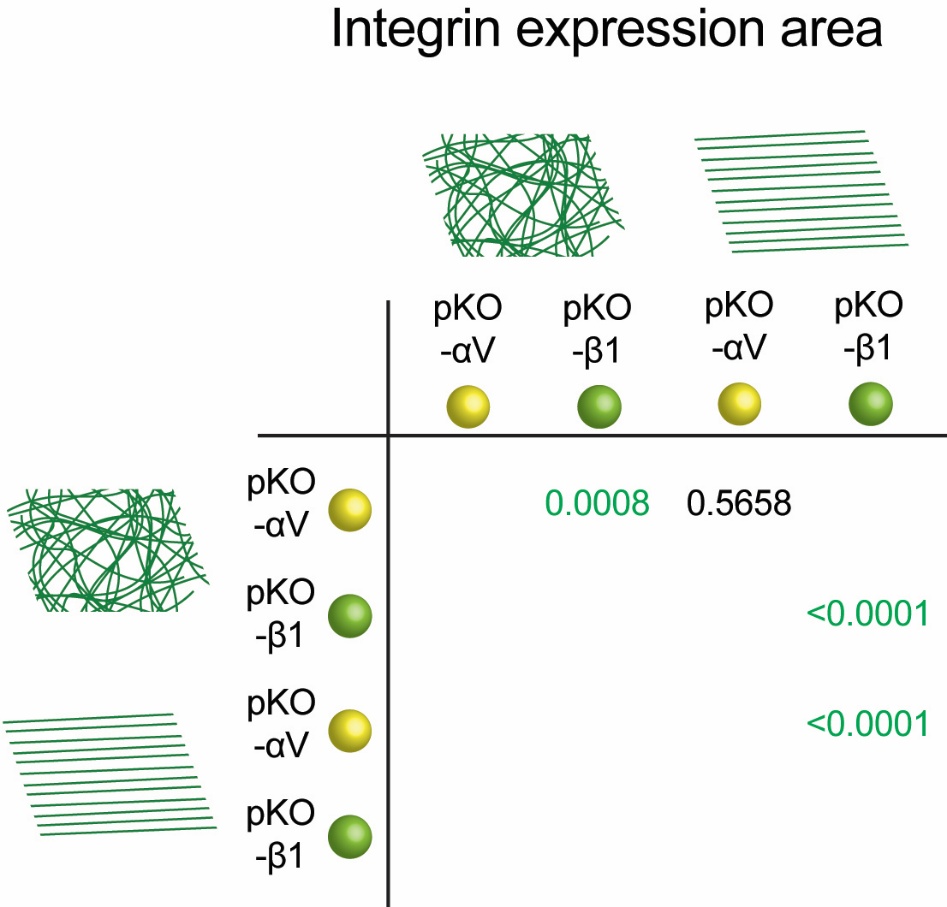


**Figure S6.** Statistical analysis of integrin expression area data in Figure 3b. *P* values were calculated using the two-tailed Mann-Whitney test. *P* values lower than 0.05 (green) are considered significant.


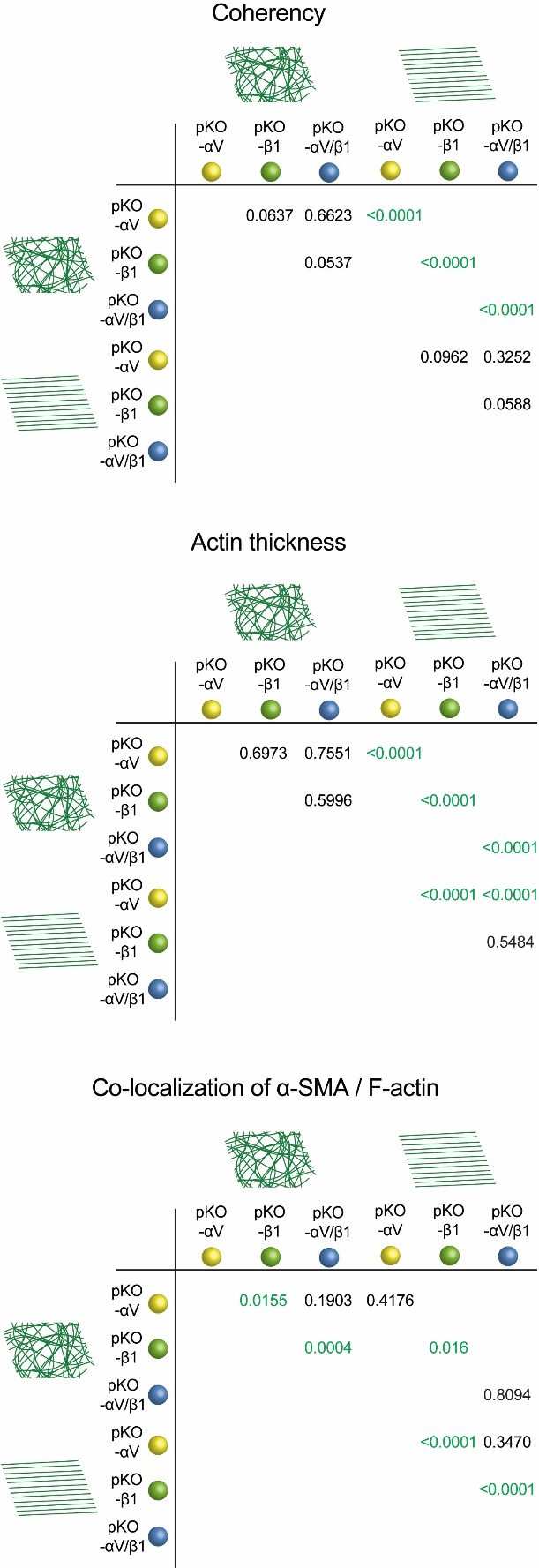


**Figure S7.** Statistical analysis of coherency, actin thickness, and α-SMA/F-actin co-localization data in Figure 4b, 4d, and 4e, respectively. *P* values were calculated using the two-tailed Mann-Whitney test. *P* values lower than 0.05 (green) are considered significant.


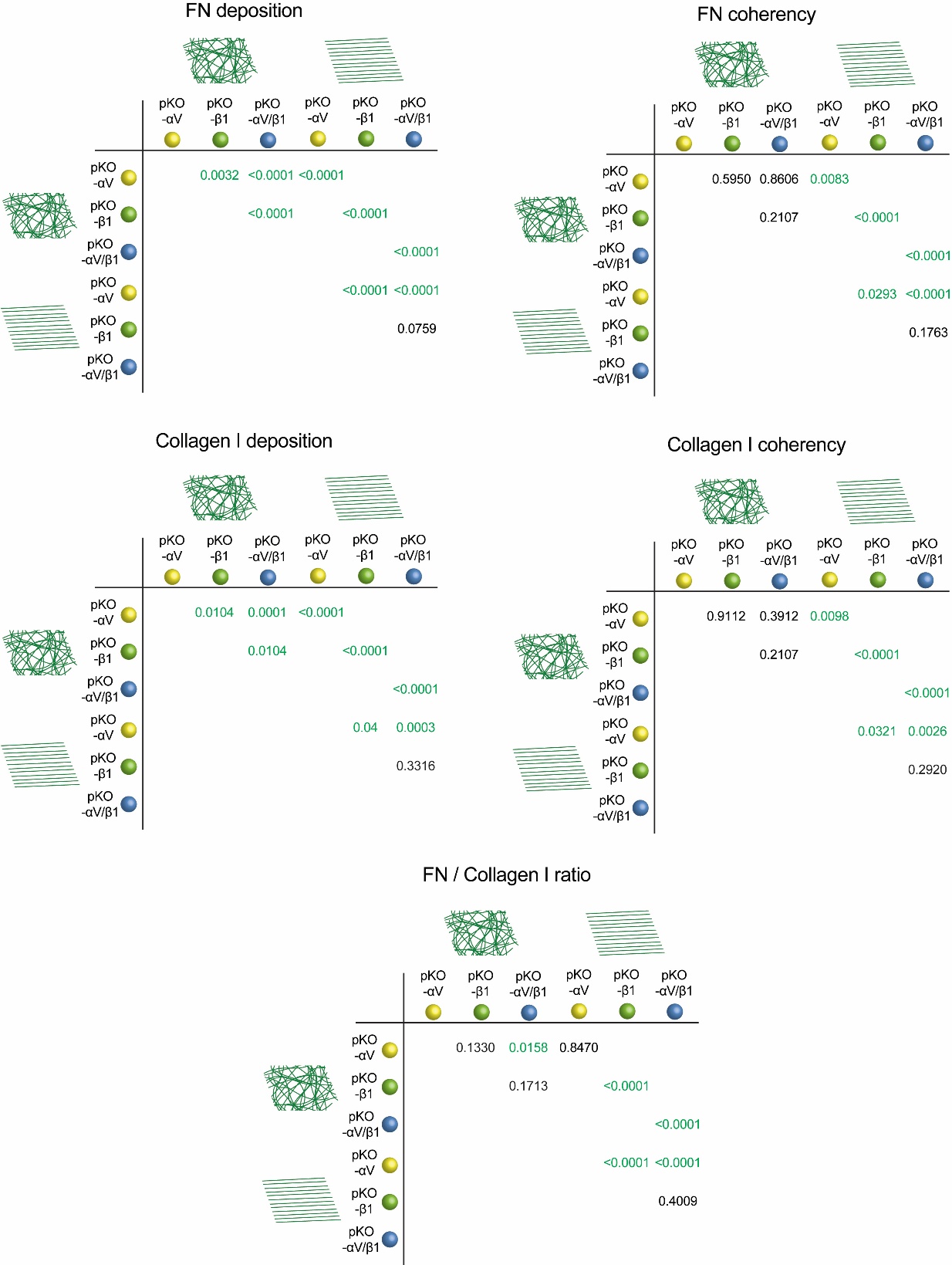


**Figure S8.** Statistical analysis of FN deposition, FN coherency, Collagen I deposition, Collagen I coherency, and FN/Collagen I ratio data in Figure 5b-f. *P* values were calculated using the two-tailed Mann-Whitney test. *P* values lower than 0.05 (green) are considered significant.


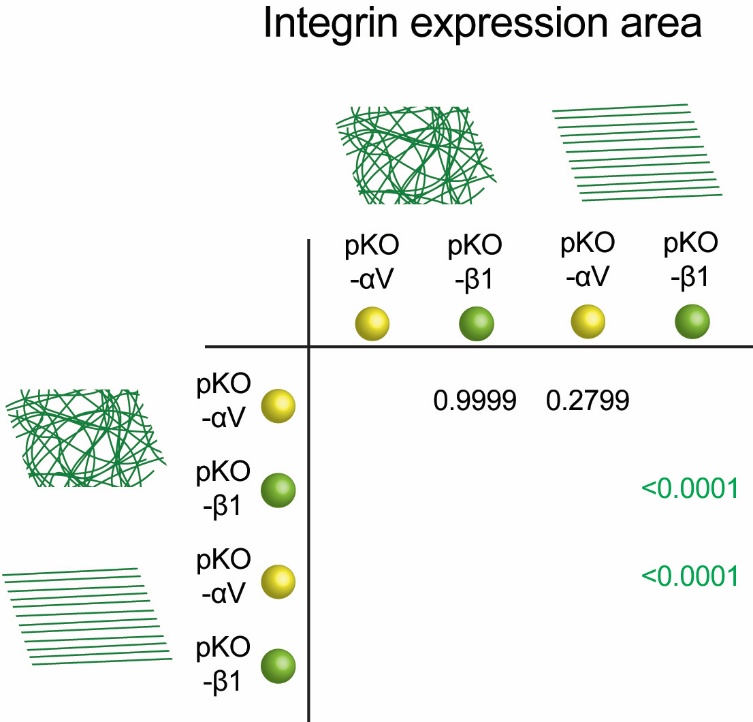


**Figure S9.** Statistical analysis of integrin expression area data in Figure 6b. *P* values were calculated using the two-tailed Mann-Whitney test. *P* values lower than 0.05 (green) are considered significant.
